# Electrode Potential-Dependent Studies of Protein Adsorption on Ti_6_Al_4_V Alloy

**DOI:** 10.3390/molecules28135109

**Published:** 2023-06-29

**Authors:** Belma Duderija, Alejandro González-Orive, Christoph Ebbert, Vanessa Neßlinger, Adrian Keller, Guido Grundmeier

**Affiliations:** 1 Technical and Macromolecular Chemistry, Paderborn University, Warburger Str. 100, 33098 Paderborn, Germany; 2 Department of Chemistry, Materials and Nanotechnology Institute, University of La Laguna, Avda, Astrofisico Francisco Sánchez s/n, 38206 San Cristóbal de La Laguna, Spain

**Keywords:** BSA, lysozyme, electrochemical conditioning, adsorption upon applied potential, surface interactions, corrosion protection

## Abstract

This article presents the potential-dependent adsorption of two proteins, bovine serum albumin (BSA) and lysozyme (LYZ), on Ti_6_Al_4_V alloy at pH 7.4 and 37 °C. The adsorption process was studied on an electropolished alloy under cathodic and anodic overpotentials, compared to the open circuit potential (OCP). To analyze the adsorption process, various complementary interface analytical techniques were employed, including PM-IRRAS (polarization-modulation infrared reflection-absorption spectroscopy), AFM (atomic force microscopy), XPS (X-ray photoelectron spectroscopy), and E-QCM (electrochemical quartz crystal microbalance) measurements. The polarization experiments were conducted within a potential range where charging of the electric double layer dominates, and Faradaic currents can be disregarded. The findings highlight the significant influence of the interfacial charge distribution on the adsorption of BSA and LYZ onto the alloy surface. Furthermore, electrochemical analysis of the protein layers formed under applied overpotentials demonstrated improved corrosion protection properties. These studies provide valuable insights into protein adsorption on titanium alloys under physiological conditions, characterized by varying potentials of the passive alloy.

## 1. Introduction

Protein adsorption on different metallic surfaces has been extensively investigated in the last 50 years [1]. Due to their specific properties such as surface passivation, which promotes enhanced corrosion resistance, low rate of metal ion release, good overall mechanical properties, and little tendency to cause adverse cell or tissue reactions, titanium, and Ti-alloys, Ti_6_Al_4_V, in particular, are widely used in surgical instruments and medical implants, especially for orthopedic and orthodontic applications [2]. The native surface oxide layer on Ti and its alloys allows tissues, proteins, and other biomolecules to interact with the surface of the biomaterial at the interface [3]. The surface chemistry as well as the structure and topography of the alloy determines the interactions between biomolecules and the biomaterial [3]. Indeed, it has been reported that the failure of an implant occurs at the interface resulting in micro-motion, inflammation, and, possibly, bone fracture [4]. To overcome these issues and ensure implant longevity, the interface between tissues and biomaterials could be optimized via smart tailoring of the surface. Physicochemical properties of metal and metal oxides formed at the surface of the Ti_6_Al_4_V biomaterial upon exposure to environmental conditions are expected to strongly influence the interaction of proteins with metal surfaces. The roughness and topography of the surface are well known to have a significant impact on cell and tissue adhesion and ultimately on the shear strength at the bone-implant interface [5]. Several different surface treatments such as polishing, anodization, and chemical etching, among others, have been largely employed to reach suitable surface conditions promoting osseointegration [6,7,8,9,10]. It has been proposed by Birch et al. [7] that by electropolishing Ti substrates an extremely smooth, leveled, reformed, and uniform TiO_2_ surface can be formed since the etching rate at the peaks or burrs is higher than that in depressions on the surface [11]. Changes in surface chemistry for Ti-rich alloys may be more complex, but, in general, electropolishing is expected to modify the oxide film and decrease the surface roughness. In the past, highly corrosive electrolytes [12] were used to perform electropolishing, which raises environmental and safety concerns. Kern et al. have shown that Ti and Ti-based alloys can be electrochemically etched or polished using less corrosive sulphuric acid–methanol electrolytes [13].

The factors that influence and determine protein adsorption on metallic surfaces are generally classified into three groups: the nature of the protein (i), the surface characteristics (ii), and the type of medium (iii). Structural flexibility [14], isoelectric point [15], and molecular size [16] of proteins as well as the surface area occupied by the adsorbed protein molecule directly affect the extent and strength of the adsorptive interactions between a protein molecule and a metal surface. Further, the pH of the adsorption medium, the electrolyte type, and the concentration naturally affect the ionization state of a protein and the charge of the solid surface, which both have a significant impact on protein adsorption behavior [16]. The surface charge is largely considered to be one of the factors that affect the protein adsorption behavior [17]. In fact, it should be possible to alter the protein adsorption processes by applying an anodic or cathodic overpotential. However, these effects are not well understood yet. Beykal et al. demonstrated that electrostatic interactions play a key role in total mass adsorption and the rate of adsorption of bovine serum albumin (BSA) on charged and uncharged gold surfaces [18]. Brusatori et al. observed that the rate and extent of albumin and cytochrome c adsorption on indium-tin-oxide coated waveguide sensor chips and a modified optical waveguide lightmode spectroscopy (OWLS) biosensing system were significantly increased by an applied voltage [19]. In fact, most studies have been carried out on model surfaces [20,21,22,23], but very little effort has been put on the investigation and understanding of the influence of the electrode potential on the electrostatic interactions of proteins, or other amphoteric macromolecules, with clinically relevant biomaterials such as Ti_6_Al_4_V.

In this article, we present the investigation of the adsorption of two different proteins, i.e., BSA and lysozyme (LYZ), on Ti_6_Al_4_V alloy at open circuit potential (OCP) and after applying an electrode potential. Prior to their usage, Ti_6_Al_4_V substrates are electrochemically polarized with the aim to get a smooth, thin, and reproducible oxide layer [7,11]. The surface characterization before and after the electrochemical treatment was carried out by X-ray photoelectron spectroscopy (XPS), Raman spectroscopy and atomic force microscopy (AFM) measurements. To investigate the effect of the electrode potential on protein adsorption, several surface analytical techniques, such as XPS, AFM, and polarization-modulation infrared reflection-absorption spectroscopy (PM-IRRAS), were employed. In situ electrochemical PM-IRRAS and electrochemical quartz crystal microbalance (E-QCM) measurements were used for the analysis of the adsorption kinetics of the two different proteins under applied potentials. BSA and LYZ were chosen as model globular protein since they are commonly present in body fluids and exhibit a quite different size and isoelectric point (pI). Considering their corresponding pIs (pI(BSA) = 4.5, pI(LYZ) = 9) [14], negative and positive overpotentials were applied to promote the adsorption of LYZ and BSA, respectively.

## 2. Results and Discussion

Net charge and size are expected to have a noticeable impact on protein adsorption. BSA and LYZ showing rather different isoelectric points (pI), namely, 4.9 and 11.4 [24], have been selected herein since they will be negatively and positively charged in PBS at pH 7.4, respectively. Consequently, with the aim to promote protein adsorption on Ti_6_Al_4_V alloy, positive and negative overpotentials were applied to the alloy in BSA or LYZ-containing PBS electrolyte.

Specifically, +0.25 and +0.5 V (vs. OCP) in 0.1, 2.0, and 4.0 g/L BSA-containing PBS solution and −0.25 and −0.5 V (vs. OCP) in 0.1, 2.0, and 4.0 g/L LYZ-containing PBS solution were applied for 2 h and pH 7.4.

Open circuit potential (OCP) curves registered for electrochemically polished Ti_6_Al_4_V alloy in the solutions of PBS with and without BSA and LYZ for 2 h at pH 7.4 and 37 °C are presented in Figure S1 in the Supplementary Materials. Cyclic voltammetry curves of electrochemically polished Ti_6_Al_4_V alloy in PBS solution without and with BSA/LYZ concentrations of 4 g/L are shown in Figure S2 in the Supplementary Materials. The course of the cyclic voltammetry curves indicated that the adsorption of BSA and LYZ at cathodic and anodic overpotentials was performed in a potential region where charging of the electric double layer dominates, and Faradaic currents can be neglected.

### 2.1. Atomic Force Microscopy (AFM)

**Figure 1:**
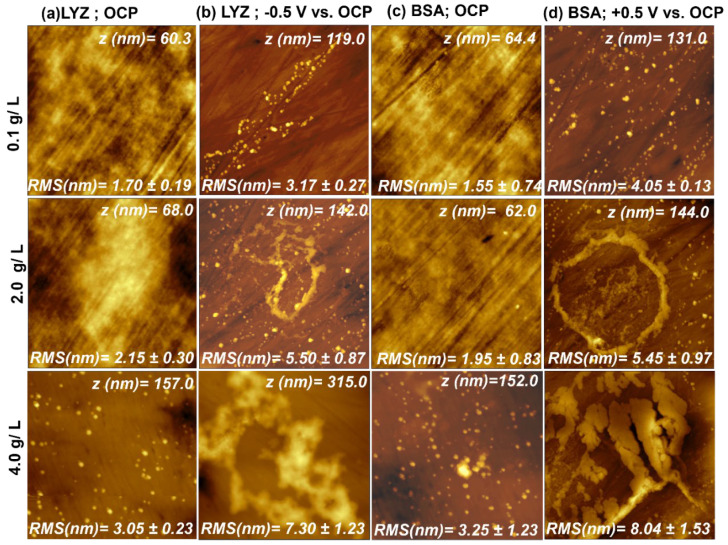
This figure shows 5.0 × 5.0 µm^2^ AFM images of (**a**) after LYZ adsorption at OCP (0.33 V_Ag/AgCl_); (**b**) after LYZ adsorption at −0.5 V (vs. OCP (0.26 V_Ag/AgCl_); (**c**) after BSA adsorption at OCP (0.33 V_Ag/AgCl_); and (**d**) after BSA adsorption at +0.5 V (vs. OCP (0.25 V_Ag/AgCl_). Images are shown for different protein concentrations.

AFM measurements were carried out to characterize the topography of the Ti_6_Al_4_V samples before and after BSA and LYZ adsorption at OCP and under an applied potential. Figure 1 shows the morphological changes on Ti_6_Al_4_V surfaces after protein adsorption.

After immersing the alloy in the protein solutions of different concentrations for 2 h, without and under applied potential, an increase in the root-mean-square (RMS) roughness of the surface could be observed for both when an overpotential was applied. This result indicates that for the negatively charged BSA, the adsorption is promoted by the positive overpotential, while the positively charged LYZ is attracted by the negative charge on the substrate surface.

Furthermore, after protein adsorption for 2 h and 37 °C at OCP for LYZ protein concentrations of 0.1 and 2.0 g/L, network-like structures could be detected with a small increase in the RMS value when compared to that exhibited by the bare alloy (see Figure S3 in the Supplementary Materials). At 4 g/L, the arising of LYZ 3D aggregates could be detected with an increase in the RMS to 3.05 ± 0.23 nm. LYZ adsorption under an applied potential of −0.5 V (vs. OCP) shows 3D aggregates even at the lowest concentration of 0.1 g/L with the RMS value of 4.05 ± 0.13 nm. When increasing the concentration from 0.1 g/L to 2.0 and 4 g/L LYZ, the amount, size, and surface coverage of 3D aggregates as well as the RMS roughness increase. The aggregates start to adsorb on top of each other and very close to others and finally collapse into a multilayered network-like structure. A very similar trend could be detected for BSA adsorption. BSA adsorption at OCP shows, at the highest concentration, the formation of 3D aggregates randomly distributed on the surface of the alloy. The RMS roughness increased slightly with BSA concentration. Further, when the BSA adsorption occurred under applied positive overpotential, the 3D aggregates were formed even at the lowest BSA concentration. With increasing concentration, the amount of the aggregates rose, forming higher network-like structures by adsorption on top of each other. The RMS roughness increased from 1.46 ± 0.14 nm for the bare alloy to the 8.04 ± 1.53 nm registered after adsorption under applied overpotential in 4 g/L of BSA. Protein adsorption at OCP is driven by a gain in entropy and the formation of attractive non-covalent interactions such as van der Waals forces and hydrogen bonds of the BSA and LYZ hydration shell [23]. During adsorption, BSA and LYZ molecules could also form an arrangement induced by electrostatic interactions between negative and positive patches in the protein known as polar ordering [23] or enclose counterions leading to the formation of growing multilayers as can be seen in the AFM images of Figure 1c,d. According to the described results, the overpotential applied to the electrode leading to the opposite surface charge increased the BSA and LYZ adsorption and subsequent growth of protein films on the Ti_6_Al_4_V alloy. This phenomenon can be associated with the attraction between negatively charged BSA or positively charged LYZ at pH 7.5 and the oppositely charged surface.

### 2.2. Polarization-Modulation Infrared Reflection-Absorption Spectroscopy (PM-IRRAS)

**Figure 2:**
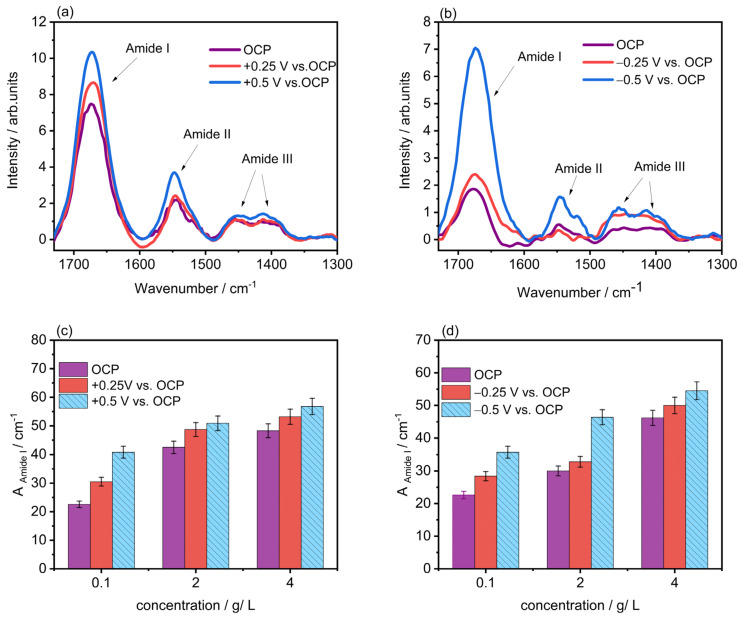
PM-IRRA spectra after BSA (**a**) or LYZ (**b**) adsorption at OCP (0.33 V_Ag/AgCl_) or under applied potential in PBS containing 4 g/L BSA or LYZ. Bar plots showing the area after peak integration of amide I band for BSA adsorption at OCP and upon applying +0.25 and +0.5 V (vs. OCP (0.24 V_Ag/AgCl_)) in PBS containing 0.1, 2.0, and 4.0 g/L in BSA (**c**), and LYZ adsorption at OCP and upon applying −0.25 and −0.5 V (vs. OCP (0.25 V_Ag/AgCl_)) in PBS containing 0.1, 2.0, and 4.0 g/L in LYZ (**d**).

PM-IRRAS analysis of electrochemically polished Ti_6_Al_4_V specimens after BSA or LYZ adsorption at OCP or under applied potential was carried out to analyze the differences in intensities of amide I, II, and III bands, characteristic of proteins [25], which are detected after their adsorption on metal surfaces. Figure 2 shows the PM-IRRA spectra of BSA or LYZ adsorption at OCP and after applying different overpotentials, +0.25 V and +0.5 V (vs. OCP) for BSA or −0.25 V and −0.5 V (vs. OCP) for LYZ, for three different concentrations.

Figure 2a,b shows PM-IRRA spectra collected after BSA or LYZ adsorption at OCP or under applied potential in PBS containing 4 g/L BSA or LYZ, respectively. The spectral region of interest includes the amide I, amide II, and amide III bands. These bands, characteristic for all proteins after adsorption on metal surfaces [26], arise from the different vibrational modes of the polypeptide backbone of the protein. The amide I band is located approximately between 1600 and 1700 cm^−1^ and represents mainly 80% of the C=O stretching vibration of the amide group coupled to 10% C-N stretching and 10% in-plane N-H bending modes [27]. The amide II band is located approximately between 1500 and 1600 cm^−1^ representing mainly N-H bending coupled to some C-N stretching of the protein’s amide groups [28]. The amide III appears between 1350 and 1480 cm^−1^ and represents C-N stretching, N-H bending, C=O stretching, and O=C-N bending [28]. In this study, the analysis was focused on the amide I band of the PM-IRRA spectra due to its strong, well-defined signal. In addition, the increase in the intensity of the amide bands has been largely associated with an increasing protein concentration on the surface [29]. The rise in the intensity of the amide bands observed in Figure 2 confirms that the larger the opposite charge on the alloy surface, the higher the amount of protein adsorbed. To further clarify this effect, bar plots of the area of amide I band after peak integration for three different concentrations of BSA or LYZ at OCP and two different potentials (vs. OCP) are presented in Figure 2c,d.

### 2.3. X-ray Photoelectron Spectroscopy (XPS)

To obtain valuable information about the chemical composition of the protein-modified Ti_6_Al_4_V surfaces, XPS measurements were performed on the bare substrates before and after BSA and LYZ adsorption both at OCP and for applied overpotentials. Electrochemically promoted protein adsorption was confirmed by the appearance of the corresponding Ti, O, C, and N signals. Survey spectra of electrochemically polished Ti_6_Al_4_V before and after protein adsorption at OCP and under applied overpotential are presented in Figures S4 and S5 in the Supplementary Materials. Results obtained from their quantitative analysis are summarized in Table 1.

**Figure 3:**
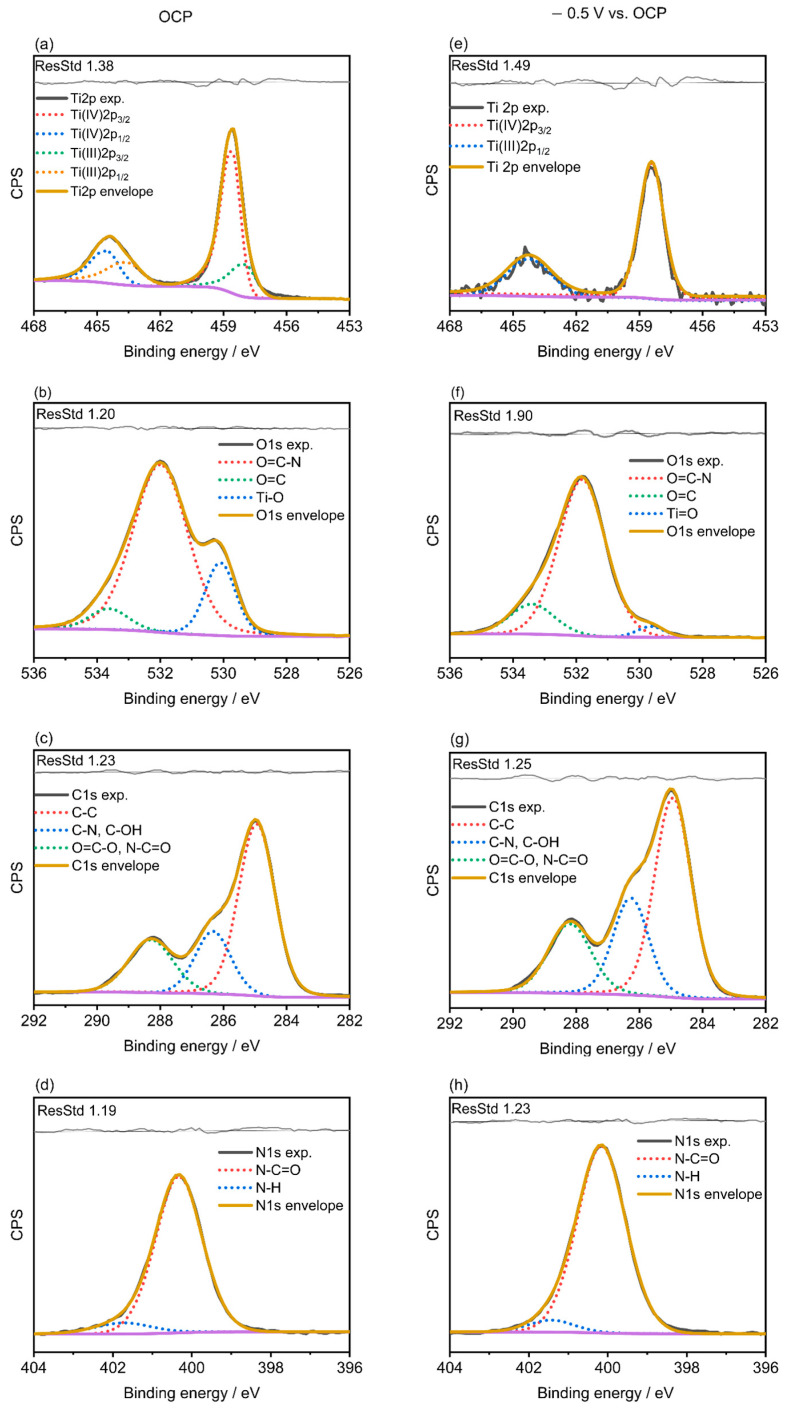
High-resolution core-level XPS spectra of Ti2p, O1s, C1s, and N1s registered after adsorption in 4 g/L of LYZ at OCP (0.33 V_Ag/AgCl_) (**a**–**d**) or after applying −0.5 V vs. OCP (0.27 V_Ag/AgCl_) (**e**–**h**) for t = 2 h at 37 °C.

**Figure 4:**
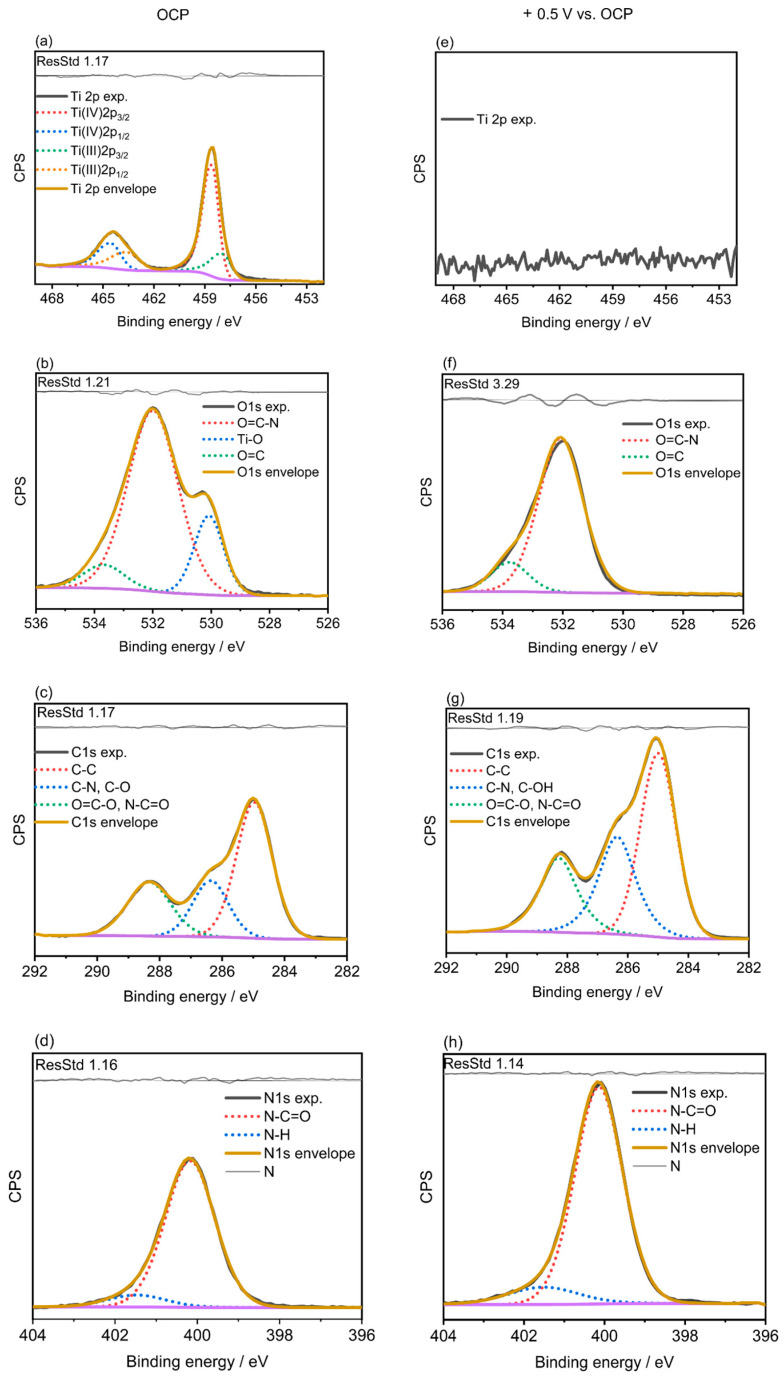
High-resolution core-level XP spectra of Ti2p, O1s, C1s and N1s registered for adsorption in 4 g/L of BSA at OCP (0.31 V_Ag/AgCl_) (**a**–**d**) or after applying +0.5 V vs. OCP (0.23 V_Ag/AgCl_) (**e**–**h**) for t = 2 h at 37 °C.

The intensity of the N1s peak of the samples immersed in the PBS solution containing BSA or LYZ was significantly higher under applied potential (see Table 1), confirming the adsorption of higher amounts of proteins. A deeper characterization of the surface chemistry for bare electrochemical polished Ti_6_Al_4_V substrates and Ti_6_Al_4_V specimens after BSA and LYZ protein adsorption with and without applied overpotential was carried out by means of the assessment of the high-resolution core level spectra of Ti2p, O1s, C1s, and N1s and is presented in Figure 3 for LYZ and Figure 4 for BSA.

Figure 3a and Figure 4a show the Ti2p high-resolution spectra for the result of protein adsorption in 4 g/L of LYZ or BSA at OCP on electrochemically polished Ti_6_Al_4_V specimens, respectively, while the ones registered under applied potential are displayed in Figure 3e and Figure 4e. The peak at 458.8 eV was attributed to Ti(IV) 2p_3/2_ and the peak at 463.4 eV to Ti(IV) 2p_1/2_. The contributions at 458.3 and 464.5 eV are assigned to Ti(III) 2p_3/2_ and Ti(III) 2p_1/2_, respectively [30]. The high-resolution spectra of Ti2p of the electrochemically polished Ti_6_Al_4_V after BSA or LYZ protein adsorption at OCP, Figure 3a and Figure 4a, exhibit similar features to those shown by the bare electrochemically polished samples displayed in Figure S6 in the Supplementary Materials by showing the characteristic peak at 458.7 eV of Ti(IV) 2p_3/2_ and the peak at 463.4 eV of Ti(IV) 2p_1/2_. The Ti(II) and Ti(III) contributions, present in the bare electrochemically polished Ti_6_Al_4_V specimen, were, however, not detected after protein adsorption under applied potentials, see Figure 3e and Figure 4e. Interestingly, the absence and the significantly reduced intensity of the Ti2p signal after BSA and LYZ adsorption, respectively, under applied potential is indicative of the growth of a thicker protein film under these experimental conditions, as can be seen in Table 1. The O1s region registered after BSA or LYZ adsorption was fitted to three contributions at 530.1 and 531.4 eV, which account for Ti-O and C=O bonds [31], respectively. Moreover, a peak at 532.1 eV, which represents the binding energy of O1s in CONH_2_ [32], was found for the sample after BSA or LYZ adsorption at OCP or after applying a potential across the interface. What is more, the Ti-O peak was noticeably reduced after adsorption in 4 g/L LYZ under applied potential and it was not found for the case of BSA (see Figure 3f and Figure 4f). These results are indicative of the adsorption of LYZ or BSA on the Ti_6_Al_4_V surface and the formation of thicker protein films for the applied overpotentials. The C1s region, Figure 3c,g and Figure 4c,g, was fitted to three contributions at 284.9, 286.4, and 288.1 eV attributed to C-C, (C-N, C-O), and (O=C-O, N-C=O), respectively. It has been reported that high-binding energy signals are caused by carbon in C-N, C-O, and O=C-N groups of the BSA and LYZ molecules [31,32]. The peak assigned to O=C-N and C-N after potential-controlled protein adsorption is significantly higher than for the alloy after protein adsorption at OCP. This indicates that higher amounts of BSA or LYZ are adsorbed on the Ti_6_Al_4_V samples when the adsorption occurs for the oppositely charged alloy surface. The N1s regions for all the samples mostly show a contribution at 400.0 eV, with a little contribution at 401.7 eV, which are attributed to N-C=O and N-H groups, respectively, which are characteristic of proteins [32], as can be seen in Figure 3d,h and Figure 4d,h.

### 2.4. Electrochemical Polarization-Modulation Infrared Reflection-Absorption Spectroscopy (E-PM-IRRAS) and Electrochemical Quartz Crystal Microbalance (E-QCM)

**Figure 5:**
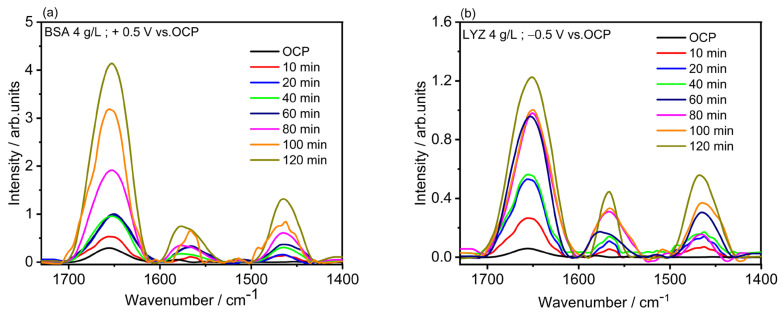
In situ PM-IRRAS measurement of time-dependent adsorption in PBS containing (**a**) 4 g/L in BSA upon applying a potential of +0.5 V (vs. OCP (0.25 V_Ag/AgCl_) and (**b**) 4 g/L in LYZ under applying −0.5 V (vs. OCP (0.27 V_Ag/AgCl_) for t = 2 h at 37 °C.

Figure 5 shows the in situ measurements carried out using the electrochemical PM-IRRAS liquid cell described in the Section 3. The spectra are showing the characteristic peaks corresponding to the amide I, II, and III bands of BSA and LYZ adsorption at 1650 cm^−1^, 1575 cm^−1^, and 1450 cm^−1^, respectively. The use of D_2_O leads to a shift of amide I band for around 18 cm^−1^ and amide II band for around 30 cm^−1^ towards lower wavenumbers, for measurement in solution compared to the measurement in air. This effect is primarily attributed to the hydrogen/deuterium exchange (H/D exchange) [33]. The applied potential does not cause any significant change in the shape of the amide bands. The increase in the intensity of the amide I band indicates that adsorption of BSA or LYZ molecules under applied potential increases with time. Figure 5a shows that after 10 min of adsorption of BSA, a significant peak of amide I could be detected with a steady increase between 20 and 40 min. Figure 5b shows a time dependent LYZ adsorption under applied potential. The adsorption of LYZ shows a similar trend with a time intensity increase of the amide I band.

**Figure 6:**
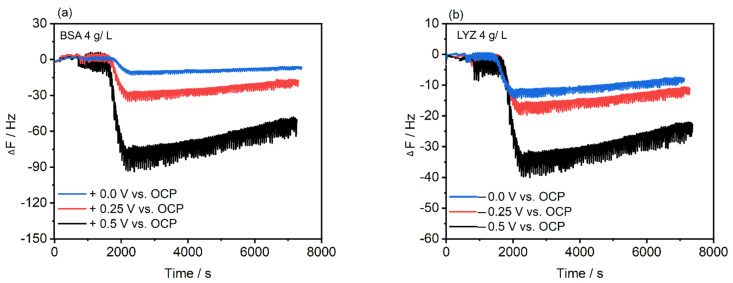
Frequency shift (*n* = 1) responses resulting from protein adsorption on TiO_2_-coated QCM sensors in PBS containing (**a**) 4 g/L BSA at OCP (0.15 V_Ag/AgCl_) or after applying +0.25 V and +0.5 V (vs. OCP (0.12 V_Ag/AgCl_) and (**b**) 4 g/L LYZ at OCP (0.15 V_Ag/AgCl_) or after applying −0.25 V and −0.5 V (vs. OCP (0.13 V_Ag/AgCl_) for t = 2 h at 37 °C.

The in situ PM-IRRAS measurements of BSA and LYZ protein adsorption confirmed that enhanced protein adsorption occurs for oppositely charged surfaces as a consequence of the respective polarization. In order to support the PM-IRRAS results and to understand how the proteins interact with the electrochemically polished Ti_6_Al_4_V alloy surface, we monitored the adsorption of BSA and LYZ using an E-QCM device with TiO_2_-coated quartz crystals. Proteins, in general, firstly adsorb onto a surface in their native conformation and then, depending on the protein-surface interaction, remain in their native conformation or unfold and spread on the surface [26,34,35]. Figure 6 shows the representative time-resolved E-QCM data showing frequency shifts registered in 4 g/L BSA or LYZ after applying two different electrode potentials, displaying characteristic time-frequency dependent profiles in response to interactions between the proteins and the surface. Upon protein injection at t = 2000 s, a marked decrease in ΔF was observed at OCP for both proteins corresponding to rapid adsorption marked by a steep drop reaching a minimum corresponding to surface saturation followed by a slow monotonic increase in ΔF, attributed to thermal drift [35].

A stronger decrease in the frequency shifts after applying potential is observed for BSA and LYZ adsorption when compared to those registered at OCP. At pH = 7.5, BSA is negative while LYZ is positively charged. When the sensor is oppositely charged, we expected to measure higher protein adsorption on the surface due to promoted electrostatic attraction. This is confirmed in Figure 6 where the measured frequency shifts (ΔF) are larger after increasing the applied potential values. Thus, the larger the overpotential is applied the higher the mass of the adsorbed protein. Figure 6a shows a drop in ΔF from −0.8 to −30 Hz after applying +0.25 V (vs. OCP) and an enhanced decrease in ΔF, i.e., from −0.9 to −93 Hz, after applying +0.5 V (vs. OCP) for BSA adsorption. In the case of LYZ adsorption, Figure 6b, a frequency shift drop occurs from −0.4 to −21 Hz after applying −0.25 V (vs. OCP) and from −0.6 to −35 Hz when −0.5 V (vs. OCP) is applied. Overall, the trends observed via QCM measurements of BSA or LYZ adsorption under applied potential are in excellent agreement with those observed via AFM and PM-IRRAS.

The amino acids present in proteins can both accept and donate a proton via reaction with their amine (-NH_2_) and carboxylic acid (-COOH) functional groups, respectively. This amphoteric behavior leads to a pH-dependent change of the protein charge. At pH values lower and higher than its pI of 4.9 [36], BSA carries a positive and negative net charge, respectively. LYZ, in contrast, has a much higher pI of 11.4 [24], so that BSA and LYZ have opposite net charges between pH 4.9 and 11.4. At pH 7.4, BSA and LYZ are in the normal form (N-form) with the most compact conformation [37,38]. Protein adsorption performed at OCP suggests that the adsorption process is driven by a gain in entropy and attractive non-covalent interactions such as van der Waals forces and hydrogen bonds of the BSA and LYZ hydration shell [25]. At OCP, BSA and LYZ molecules could also form an arrangement induced by electrostatic interactions between negative and positive patches in the protein known as polar ordering [25] or enclose counterions leading to the formation of a multilayer growing that could be seen in the AFM images of Figure 1a,c. According to the described results, the potential applied to the electrode increased the BSA and LYZ adsorption and growth on the Ti_6_Al_4_V alloy. However, an electrically polarized surface would naturally attract or repel protein molecules. An electric field-based interaction was proposed as one of the main mechanisms for the adsorption of a protein on electrode surfaces in previous studies [39,40,41,42,43]. The hypothesis is that this phenomenon can be associated with the attraction between negative BSA or positively charged LYZ at pH 7.4 and the oppositely charged surface.

### 2.5. Electrochemical Characterization

**Figure 7:**
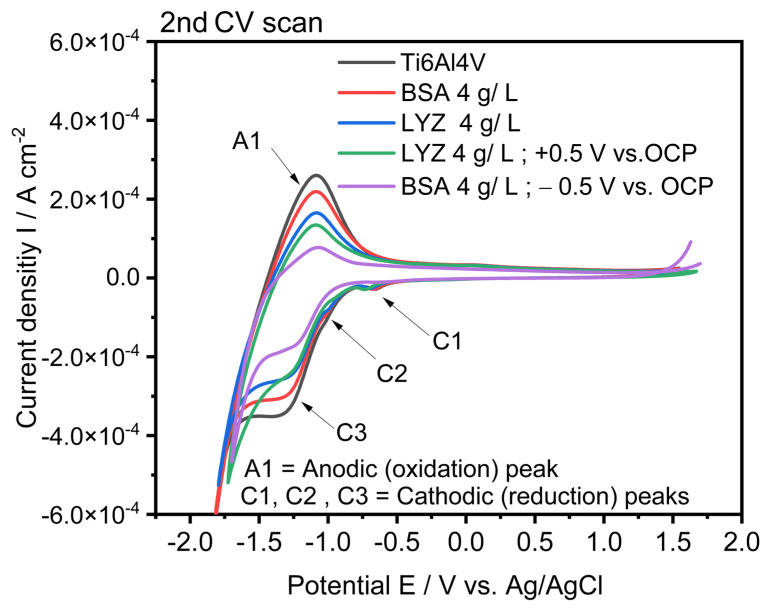
Cyclic voltammograms registered for bare Ti_6_Al_4_V alloy after electrochemical polishing (grey line) and after protein adsorption for t = 2 h and 37 °C in 4 g/L BSA at OCP (0.33 V_Ag/AgCl_ red); in 4 g/L LYZ at OCP (0.33 V_Ag/AgCl_ (blue)); in 4 g/L BSA at +0.5 V (vs. OCP (0.24 V_Ag/AgCl_) (green); and in 4 g/L LYZ at −0.5 V (vs. OCP (0.26 V_Ag/AgCl_) (magenta).

The charge transfer capabilities exerted by adsorbed BSA and LYZ on electrochemically polished Ti_6_Al_4_V surfaces without and under applied potential have been characterized by registering cyclic voltammograms in PBS (pH = 7.5). Siddiqui et al. have studied BSA adsorption on Ti3Cu alloy showing similar results to ours for the BSA adsorption on metal alloys [44]. Figure 7 shows the second cycle registered for all different samples resulting from protein adsorption plotted together. By analyzing the voltammetric peaks in the CV collected for the bare Ti_6_Al_4_V, one anodic peak A1 (corresponding to Ti (III) to Ti (IV) oxidation), and three cathodic peaks, i.e., C1 arising at −0.66 V (vs. Ag/AgCl), C2 at −0.94 V (vs. Ag/AgCl), and C3 at −1.29 V (vs. Ag/AgCl), preceding the hydrogen evolution reaction (HER) can be distinguished. A similar electrochemical profile has been reported for anatase TiO_2_ [45]. It was observed that after protein adsorption, a significant decrease in the current peaks is observed, which is indicative of the formation of thin protein films progressively blocking charge transfer through them and limiting diffusion of electrolyte ions to the metal oxide surface. Some other studies also showed that protein adsorption stops the metal ions release by blocking electrochemical dissolution and slowing down the corrosion [46,47]. The thicker the protein film adsorbed on the surface of the alloy, the smaller the voltammetric peaks. The observed inhibition of the redox peaks in the CVs correlates well with the adsorbed amounts of protein as discussed before.

Once BSA and LYZ protein film was adsorbed on the alloy, the reduction C1 and C2 (cathodic) peaks mostly disappeared as can be seen in Figure 7, and the voltammograms showed only titanium oxidation and reduction peaks associated with the Ti (III)/Ti (IV)redox couple preceding HER with a gradual decrease in current densities associated to them.

## 3. Materials and Methods

Prior to their usage, Ti_6_Al_4_V alloy plates (purchased from Goodfellow) were solvent-cleaned, i.e., ultrasonicated for 15 min in pure tetrahydrofuran (≥98%, Sigma-Aldrich, St. Louis, MO, USA), propan−2-ol (≥98%, Sigma-Aldrich), and ethanol (≥98%, Sigma-Aldrich). BSA (≥98%, Sigma-Aldrich) and LYZ (≥98%, Sigma-Aldrich) proteins were dissolved in phosphate buffer saline solution (PBS, pH = 7.4, Sigma-Aldrich) with concentrations of 0.1 g/L, 2.0 g/L, and 4.0 g/L.

### 3.1. Surface Pre-Conditioning and Electrochemical Modification of the Substrate Surfaces

Solvent-cleaned Ti_6_Al_4_V alloy plates were first ground with SiC paper (grit size 220 to 4000, WS Flex, Hermes, Paris, France) until they were mirror polished. The substrates were then rinsed in Milli-Q water and ethanol and dried under nitrogen flow. Next, the electrochemical polishing was performed in a three-electrode cell setup using Ti_6_Al_4_V plates (1 × 1 cm^2^) as working electrodes, a gold coil as the counter electrode, and a commercial Ag/AgCl (3 M KCl, Radiometer Analytical, Villeurbanne, France) as the reference electrode in an acidic dried electrolyte, 2.25 M H_2_SO_4_ (≥98%, Sigma-Aldrich) in anhydrous CH_3_OH (≥98%, Sigma-Aldrich), for 180 s at 9 V at room temperature [7]. With this system, only the portion exposed to the electrolyte, i.e., 0.785 cm^2^ of the surface, was electrochemically polished. A Reference 600^TM^ (Gamry Instruments, Warminster, PA, USA) was used to carry out the chronoamperometry (CA) experiments. The Ti_6_Al_4_V plate was placed between a copper electrical contact and the aperture, which ensured that the plates were sandwiched between two perspex blocks. The AFM, XPS, and Raman measurements of Ti_6_Al_4_V samples before and after electrochemical polishing are presented in Figures S3, S4, S6 and S7 in the Supplementary Materials, respectively. The latter confirms the successful decrease in the roughness on the nano and microscale, which is accompanied by an increase in the TiO_2_ ratio in the oxide layer.

### 3.2. Protein Adsorption

The BSA and LYZ protein adsorption at pH = 7.4 on the electrochemically polished Ti_6_Al_4_V substrates were first performed whit an ex situ approach by dipping the samples in a PBS aqueous solution containing 0.1, 2.0, and 4 g/L of BSA and LYZ, separately, for 2 h at 37 °C. Second, the potential dependent protein adsorption studies were performed in a three-electrode-cell setup equipped with an Ag/AgCl reference electrode, a gold wire counter electrode, and treated Ti_6_Al_4_V substrates as working electrodes in a 4, 2, and 0.1 g/L BSA and LYZ in PBS via chronoamperometry by applying +0.25 and +0.5 V (vs. OCP, 30 min) for 2 h at 37 °C for adsorption of BSA and −0.25 and −0.5 V (vs. OCP, 30 min) for 2 h at 37 °C for LYZ adsorption. After the measurement was completed, the samples were carefully rinsed, dropwise, with Mili-Q water and dried under gentle nitrogen flux. The as-prepared samples were then characterized with XPS, AFM, and PM-IRRAS.

### 3.3. Atomic Force Microscopy (AFM)

AFM imaging was performed using a JPK Nanowizard III (Bruker GmbH, Billerica, MA, USA) equipped with an acoustic enclosure in alternating contact mode operating in ambient air conditions at a scan rate of 0.5–1.2 Hz. HQ:NSC18/AlBS cantilevers (75 kHz and 2.8 Nm^−1^, nominal radius of 8 nm, MikroMasch, Tallinn, Estonia) were used. For subsequent data processing and calculation of RMS roughness values for estimation of surface roughness, the Gwyddion open-source software [48] (V 2.56, Gwyddion) was employed. RMS roughness measurements were obtained from the average of the roughness values collected from three different 5.0 × 5.0 µm^2^ areas present in three equally prepared samples.

### 3.4. X-ray Photoelectron Spectroscopy (XPS)

XPS measurements were performed using an Omicron ESCA+ system (Omicron NanoTechnology GmbH, Taunusstein, Germany) equipped with a hemispherical energy analyzer at a base pressure of <5 × 10^−10^ mbar. Spectra were recorded at pass energies of 100 eV for survey spectra and 20 eV for core-level element spectra. A monochromatic Al Kα (1486.3 eV) X-ray source with a spot diameter of 1 mm was used. The take-off angle of the detected photoelectrons was set to 60° regarding the surface plane. XPS spectra were internally calibrated to the C1s peak (binding energy, BE = 285.0 eV). For peak fitting, a combination of Gaussian (70%) and Lorentzian (30%) distribution was used. Data analysis was performed with CASA-XPS software. Quantification was performed through an integration of the peaks with respect to the corresponding relative sensitivity factor (RSF) values. A Shirley-type background correction was used.

### 3.5. Polarization-Modulation Infrared Reflection-Absorption Spectroscopy (PM-IRRAS)

The adsorbed protein on electrochemically polished Ti_6_Al_4_V samples was characterized by using a Vertex 70 (Bruker Optics, Ettlingen, Germany) with an external PM-IRRAS set-up (p-polarization with an aluminum wire grid), which was modulated at 50 kHz with a ZnSe Photo-Elastic-Modulator (PMA50, Bruker Optics Germany) equipped with a ZnSe lens onto a cryogenic mercury cadmium telluride (LN-MCT) detector. The total number of scans was set as 1024. The energy resolution was set as 2 cm^−1^.

### 3.6. Electrochemical Polarization-Modulation Infrared Reflection-Absorption Spectroscopy (E-PM-IRRAS)

**Figure 8:**
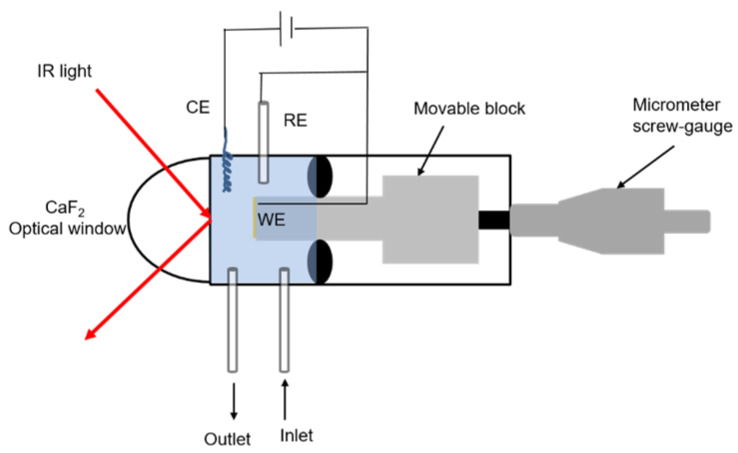
Schematic of the in situ electrochemical PM-IRRAS liquid cell.

PM-IRRAS is used to study molecular assemblies at various interfaces. The development of a liquid PM-IRRAs cell allows the monitoring of in situ molecular assembly processes at the electrolyte/metal interface. Thus, the PM-IRRAs liquid cell setup allows the investigation of thin film formation on metallic surfaces at several electrolyte conditions, such as pH, electrolyte composition, and applied potential as well as the study of specific interactions between adsorbed molecules and the sample. The schematic representation of the cell is illustrated in Figure 8.

To examine the molecular structure of an electrolyte/metal surface in more detail, the liquid cell is inserted into the IR chamber. As can be seen in Figure 1, the cell consists of a movable block, micrometer screw-gage, sample holder for the working electrode, liquid in- and outlet, optical window, and counter and reference electrodes. The movable block can be driven back and forth by means of a micrometer screw. The samples are attached to the top of the block with vacuum. To carry out the measurements using IR radiation, the substrate is placed in front of a hemicylindric CaF_2_ crystal. For optimal intensities, the detector was set at an angle of 45°. Previous studies of Brand [49] have shown that when the IR path passes through new phases, the incoming intensity to the detector is affected by electrolyte thickness and optical window material (angle of incidence). The results have shown that the electrolyte thickness of 5 μm in combination with CaF_2_ provides very good incoming intensities at the detector. The spectral range is given by the optical window material. In the case of CaF_2_, it ranges from 4000 to 1000 cm^−1^, which allows any type of metallic substrates that reflect the IR light to be investigated [49].

However, the measurements in liquid generally show lower intensities than in air. The aperture setting was set to 2 mm and the number of scans was 1024. Since applying positive and negative electrochemical potentials (vs. OCP) in the ex situ measurements via the three-electrode setup has shown enhanced adsorption of BSA and LYZ proteins from bulk solution at physiological pHs, we next studied the in situ adsorption of BSA and LYZ using the PM-IRRAS liquid cell.

For this study, the mechanically polished Ti_6_Al_4_V samples were mounted in the PM-IRRAS liquid cell in the IR chamber, and the BSA and LYZ solution prepared in PBS (pH = 7.4 in 100 mL D_2_O water at 37 °C) 4 g/L BSA and LYZ were pumped in the cell with a flow of 80 µL/min, respectively. The protein adsorption on the electrochemically polished Ti_6_Al_4_V was performed by means of a three-electrode-cell, with Ag/AgCl reference electrode, a gold wire-counter electrode, and treated Ti_6_Al_4_V substrates as working electrodes integrated into the PM-IRRAS liquid cell. For BSA and LYZ adsorption, bias potentials of +0.5 V and −0.5 V (vs. OCP) were applied, respectively. First, OCP was measured for 5 min in the protein-containing PBS solution in D_2_O. After that, the corresponding potentials (vs. OCP) were applied for 10 min and the cell was washed with PBS in D_2_O. Next, the sample was brought to the optical window (around 5 µm distance) to start the IR measurement. The same procedure was repeated after 10, 20, 40, 60, 80, 100, and 120 min for both BSA and LYZ. When finished, the samples were separated from the optical window with the micrometer screw, and the PBS solution was pumped through the cell. Next, the cell was washed with D_2_O water by pumping it through the cell for 10 min. Afterward, the cell was disassembled and the optical window was cleaned with ethanol. The use of D_2_O leads to a shift of the amide I band at around 18 cm^−1^ and the amide II band at around 30 cm^−1^ towards lower wavenumbers for measurements carried out in liquid in comparison to those performed in air. It is important to mention that the use of D_2_O is indicated when investigating proteins to avoid overlapping of the amide I signal with water peaks. The results fit very well with the literature data [49]. This effect is primarily attributed to the hydrogen/deuterium exchange (H/D exchange) and thus to the mass of the oscillating atoms and the oscillation frequency [50].

### 3.7. Electrochemical Quartz Crystal Microbalance (E-QCM)

To understand how proteins interact with the electrochemically polished Ti_6_Al_4_V alloy, we monitored the adsorption of BSA and LYZ using the E-QCM device (E-QCM 10 M; Gamry Instruments) [51]. E-QCM allows tracking the changes in the resonant frequency of the sensor, which may be related to mass changes that occur due to protein adsorption upon electrochemical control using the gold-coated 5 MHz quartz crystal as the working electrode; a platinum foil serves as the counter electrode, and a Ag/AgCl electrode as the reference electrode connected to potentiostat-galvanostat Gamry 600^TM^. To support the results acquired from the PM-IRRAS liquid cell measurement, 5 MHz quartz crystals (Quartz Pro, Sweden AB, Stockholm, Sweden) were coated with 80 nm of Ti_6_Al_4_V and 7 nm of TiO_2_. The 4 g/L BSA and LYZ solution was prepared by dissolution in 10 mM PBS. After 30 min of system stabilization, a 500 µL of BSA or LYZ solution was injected into the system with a flow rate of 80 µL/min using a peristaltic pump. The protein adsorption kinetic was monitored for 2 h using Gamry Resonator software (Gamry Instruments) at 37 °C after measuring OCP for 5 min followed by applying a potential of +0.25 and +0.5 V (vs. OCP) for BSA, while −0.25 and −0.5 V (vs. OCP) were applied for the case of LYZ.

### 3.8. Electrochemical Characterization

A three-electrode setup cell connected to a potentiostat-galvanostat Gamry 600^TM^ was used to carry out the electrochemical characterization of the substrates. Bare and protein-modified Ti_6_Al_4_V plates (1 cm × 1 cm) were used as working electrodes, whereas a gold coil and a commercial Ag/AgCl (3 M KCl, Radiometer Analytical) were employed as the counter and reference electrodes, respectively. The surface of the samples was exposed to the supporting electrolyte solution, namely, PBS at pH = 7.4 and 37 °C. The OCP was measured for 30 min. The charge-transfer properties exhibited by protein films were investigated by carrying out cyclic voltammetry measurements. Five consecutive cyclic voltammograms were registered for each sample at 50 mVs^−1^.

## 4. Conclusions

In the present study, we investigated the adsorption of negatively charged albumin (BSA) and positively charged lysozyme (LYZ) at physiological pH at OCP or under applied potential on electrochemically polished Ti_6_Al_4_V alloy, focusing on the impact of surface electrode potential and the concentration of the proteins. The protein adsorption under potential control at the electrolyte/Ti_6_Al_4_V alloy interface was demonstrated by means of complementary interface analytical techniques. Data extracted from AFM, XP, and PM-IRRA spectra proved the formation of firmly attached thin protein films on the metallic substrates, and the enhanced adsorption of BSA and LYZ on the alloy occurred under suitably applied electrode potentials. E-PM-IRRAS and E-QCM measurements demonstrated that electrostatic interactions play a key role in the total mass of BSA and LYZ adsorbed to charged surfaces by showing an increase of protein adsorption with time for increasing applied potentials. When the protein adsorption is performed at open circuit potential (OCP), BSA and LYZ adsorb on the surface of the Ti-alloy driven by attractive non-covalent interactions. If an overpotential is applied, the respective double layers lead to an additional electrostatic attraction of the oppositely charged protein. The results presented herein would open the door for the in situ monitoring of the evolution of the conformation and secondary structure of adsorbed proteins, together with the assessment of protein adsorption kinetics, on technical materials and upon electrochemical control by means of a highly sensitive surface analysis technique such as PM-IRRAS.

## Figures and Tables

**Table 1:** XPS surface composition analysis in at.-% of electrochemically polished Ti_6_Al_4_V alloy substrates after 4 g/L BSA or LYZ adsorption at OCP or under applied potential for t = 2 h at 37 °C; obtained from survey XPS spectra displayed in Figure S5 in the Supplementary Materials.

Sample	Atomic Percentages (at.-%)			
	Ti2p	O1s	C1s	N1s
LYZ at OCP	3.8	38.7	48.3	7.4
LYZ at −0.5 V vs. OCP	2.2	25.4	56.2	12.7
BSA at OCP	2.9	26.9	61.1	8.9
BSA at + 0.5 V vs. OCP	-	18.5	67.8	13.7

## Data Availability

The data presented in this study are available on request from the corresponding author.

## Supplementary Materials

The following supporting information can be downloaded at https://www.mdpi.com/article/10.3390/molecules28135109/s1; Figure S1: OCP curves registered for electrochemically polished Ti_6_Al_4_V alloy in PBS solution (black line and dots) and PBS solutions containing 4 g/L BSA (red dots) or LYZ (green) for 2 h at pH 7.4 and 37 °C. Figure S2: Cyclic voltammograms registered for bare Ti_6_Al_4_V alloy after electrochemical polishing (black line) in PBS (black line) and PBS containing 4 g/L BSA (blue) or 4 g/L LYZ (red). Figure S3: 2.0 × 2.0 µm^2^ AFM images of bare Ti_6_Al_4_V (a) and after electrochemical polishing at 9 V for 180 s (b). Topographic and phase contrast images are displayed in the upper left and right panels, respectively. Representative cross-section profiles are portrayed in the corresponding lower panels. Figure S4: XPS survey spectra registered for (a) bare Ti_6_Al_4_V and (b) after electrochemical polishing at 9 V for 180 s. Figure S5: XPS survey spectra of (a) electrochemically polished Ti_6_Al_4_V alloy (b); after adsorption of 4 g/L BSA at OCP (c); after adsorption of 4 g/L of BSA under applied potential (+0.5 V vs. OCP) (d); after 4 g/L LYZ adsorption at OCP and (e) after 4 g/L LYZ adsorption under applied overpotential (−0.5 V vs. OCP). Figure S6: High-resolution core-level XP spectra of (a) Ti2p, (b) O1s, (c) C1s, and (d) N1s registered for a bare Ti_6_Al_4_V sample after electrochemical polishing at 9 V for 180 s. Figure S7: Raman spectra of Ti_6_Al_4_V before (black line) and after electrochemical polishing at 9 V for 180 s (red).

## Sample Availability

Samples of the compounds are not available from the authors.
