# Supplementary material for: Electrode Potential-Dependent Studies of Protein Adsorption on Ti6Al4V Alloy
Source: Molecules. 2023 Jun 29;28(13):5109. doi: 10.3390/molecules28135109 (PMC10343353; doi:10.3390/molecules28135109)
Supplement: Supplementary file 1 [file molecules-28-05109-s001.zip › molecules-2443754-supplementary.pdf]

Supporting Information for

Electrode Potential dependent studies of protein adsorption on Ti6Al4V alloy

Belma Duderija,[1] Alejandro González-Orive[2*], Christoph Ebbert [1], Vanessa Neßlinger[1], Adrian Keller[1], Guido Grundmeier[1*]

[1]Technical and Macromolecular Chemistry, University Paderborn, Warburger Str. 100, 33098 Paderborn, Germany

[2]Department of Chemistry, Materials and Nanotechnology Institute, University of La Laguna, Avda. Astrofísico Francisco Sánchez s/n, 38206 San Cristóbal de La Laguna, Spain

*Correspondence: A.G.-O. (agorive@ull.edu.es) and G.G. (guido.grundmeier@uni-paderborn.de)

1. OCP transients of electropolished Ti6Al4V with and without protein-containing electrolyte

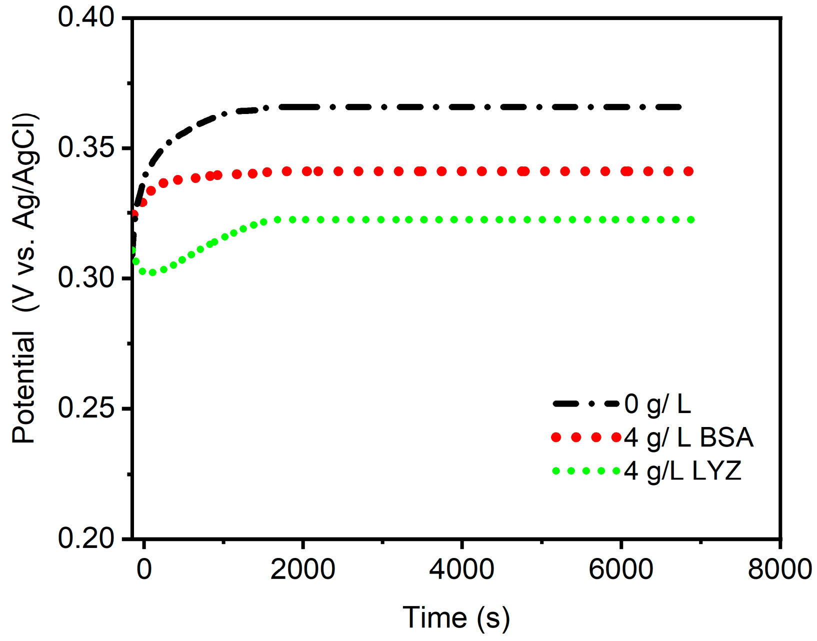

Figure S1: OCP curves registered for electrochemically polished Ti6Al4V alloy in PBS solution (black line and dots) and PBS solutions containing 4 g/L BSA (red dots) or LYZ (green) for 2 h, at pH 7.4 and 37°C.

Figure S1 shows the evolution of the open circuit potential (OCP) of electrochemically polished Ti6Al4V alloy immersed in aerated solutions of PBS containing 0 g/ L and 4 g/L BSA and LYZ as a function of time at 37 °C for 2h. The investigated samples showed similar behavior. Immediately after immersion in the PBS solution, the potential shifted anodically, reaching a stable value after about 20 minutes. The potential shift towards more positive values is a consequence of the change in the passive oxide film during immersion. The OCP values in BSA and LYZ-containing electrolytes decreased, which might be related to the effect of the protein adsorption onto the surface of the alloy which inhibits the cathodic reactions. Table S1. presents five OCP measurements of electrochemically polished Ti6Al4V alloy in aerated solutions of PBS containing 0 g/ L and 4 g/L BSA or LYZ including the calculated median and standard deviation.

Table S1. OCP values of five electrochemically polished Ti6Al4V alloy measurements in aerated solutions of PBS containing 0 g/ L and 4 g/L BSA or LYZ including the calculated median (M) and standard deviation (SD).

**Sample**	**OCP values (V vs. Ag/AgCl)**		
	**0 g/ L**	**4g/L LYZ**	**4 g/ L BSA**
1	0.37	0.31	0.34
2	0.33	0.15	0.35
3	0.34	0.27	0.28
4	0.27	0.32	0.27
5	0.35	0.29	0.31
M	0.33	0.26	0.25
SD	0.04	0.07	0.03

2. Cyclic voltammograms (CV) curves of electropolished Ti6Al4V with and without protein-containing electrolyte

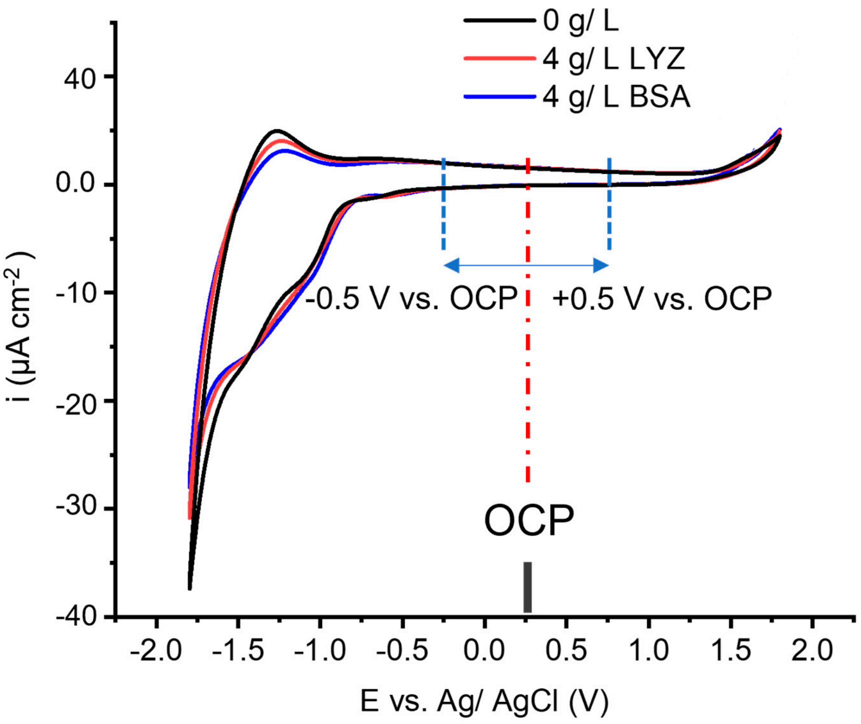

Figure S2: Cyclic voltammograms registered for bare Ti6Al4V alloy after electrochemical polishing (black line) in PBS (black line) and PBS containing 4 g/L BSA (blue) or 4 g/L LYZ (red).

Figure S2 presents the second cycle recorded for the bare Ti6Al4V alloy in PBS after electrochemical polishing (black line), in the presence of 4 g/L BSA (blue line), and in the presence of 4 g/L LYZ (red line). The voltammograms reveal some faradaic anodic and cathodic voltammetric peaks preceding the hydrogen evolution reaction (HER). The presence of proteins in the electrolyte leads to a decrease in the current peaks, indicating the formation of thin protein films that impede charge transfer and limit electrolyte ion diffusion to the metal oxide surface.

The potential-dependent protein adsorption experiments were conducted using chronoamperometry, applying +0.25 and +0.5 V (vs. OCP (0.26 $V_{Ag/AgCl}$) for 30 minutes) for 2 hours at 37 °C for BSA adsorption, and -0.25 and -0.5 V (vs. OCP (0.25 $V_{Ag/AgCl}$) for 30 minutes) for 2 hours at 37 °C for LYZ adsorption.

The cyclic voltammetry curves shown in Figure S2 demonstrate that the adsorption of BSA and LYZ through chronoamperometric measurements at cathodic and anodic overpotentials occurs within a potential range (-0.4 to 1.3 $V_{Ag/AgCl}$) dominated by charging of the electric double layer, while Faradaic currents can be neglected.

3. AFM morphological characterization of Ti6Al4V substrates

Figure S3 shows AFM images registered for a bare Ti6Al4V substrate before and after electrochemical polishing, exhibiting their characteristic topographic features. After polishing, Figure S3b, the grain boundaries and crystallographic phases, clearly delimited and more visible in the phase contrast image in Fig. S3a, disappeared, and a very homogeneous surface could be observed. A noticeable decrease in the RMS roughness, i.e., from 6.09 ± 1.51 to the 1.46 ± 0.78 nm measured after polishing, was observed, as can be inferred from the representative cross-sectional profiles as well.

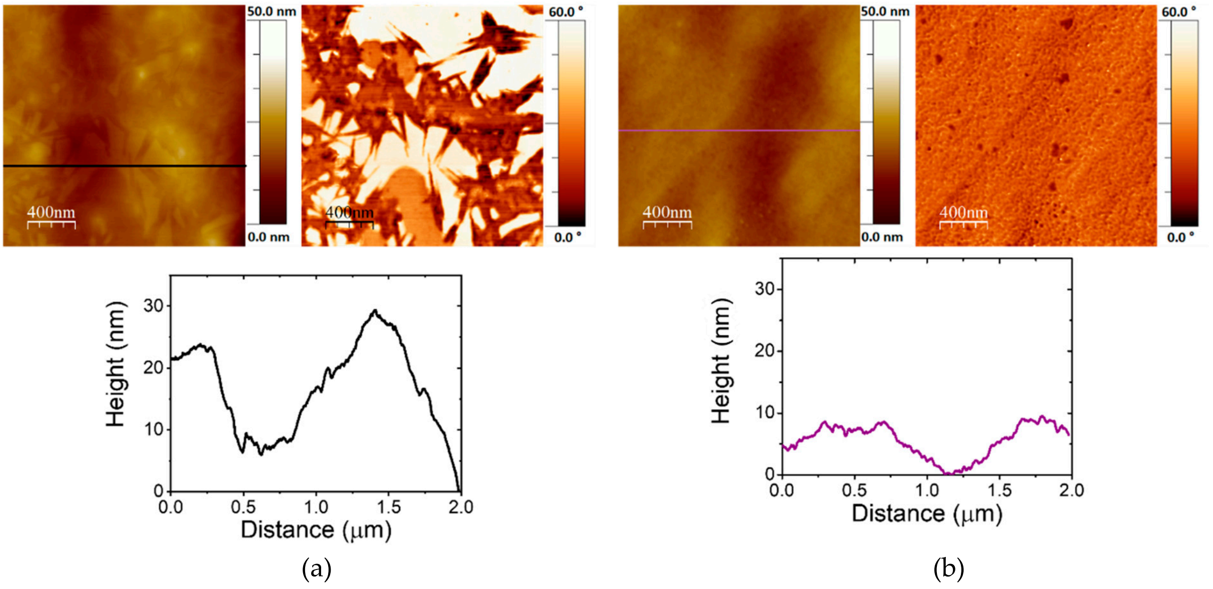

Figure S3: 2.0 x 2.0 μm² AFM images of bare Ti6Al4V (a) and after electrochemical polishing at 9 V for 180 s (b). Topographic and phase contrast image are displayed in the upper left and right panels, respectively. Representative cross section profiles are portrayed in the corresponding lower panels.

4. XPS Characterization for bare Ti6Al4V and after electrochemical polishing

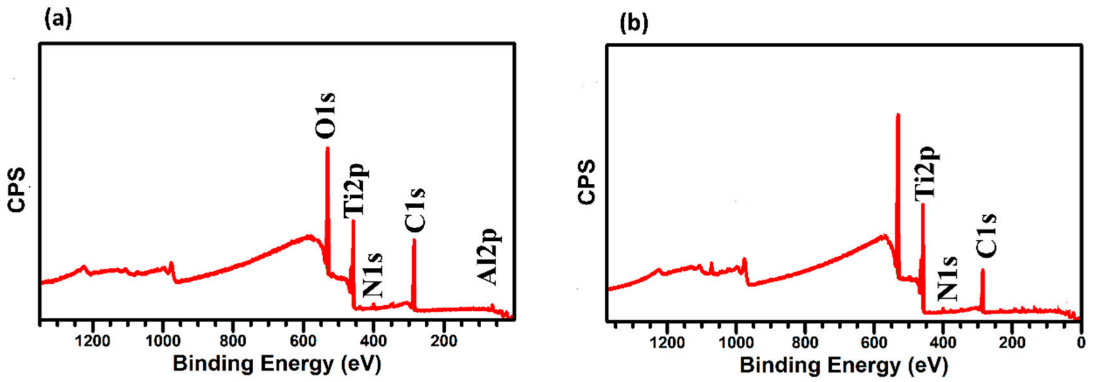

Figure S4: XPS survey spectra registered for (a) bare Ti6Al4V and (b) after electrochemical polishing at 9 V for 180s.

5. XPS Characterization after protein adsorption

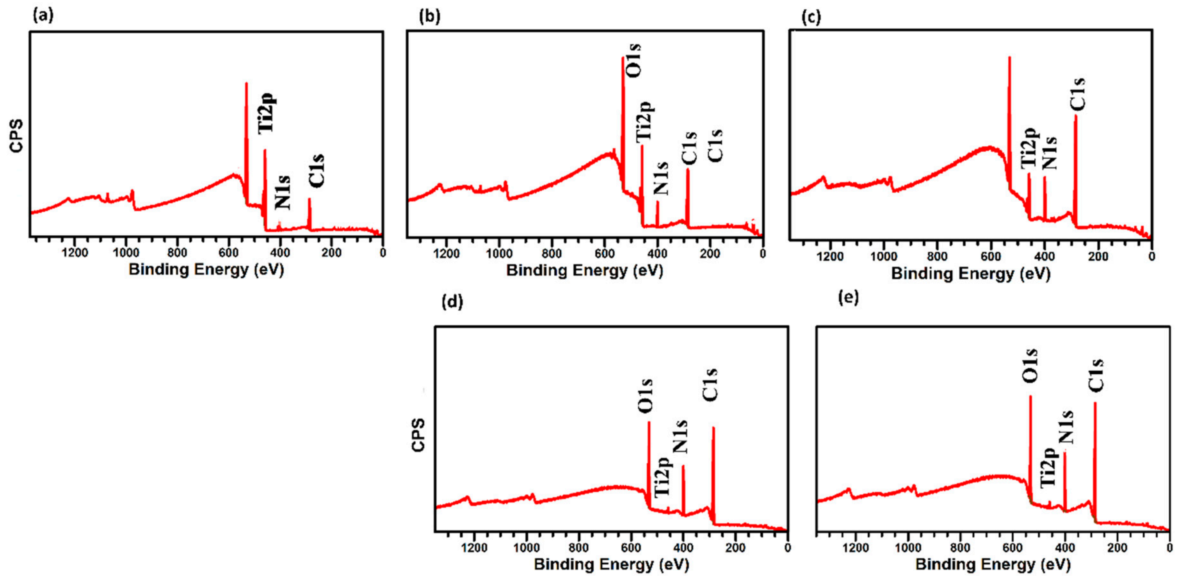

Figure S5: XPS survey spectra of (a) electrochemically polished Ti6Al4V alloy (b) after adsorption of 4 g/L BSA at OCP (c) after adsorption of 4 g/L of BSA under applied potential (+0.5 V vs. OCP) (d) after 4 g/L LYZ adsorption at OCP and (e) after 4 g/L LYZ adsorption under applied overpotential (-0.5 V vs. OCP).

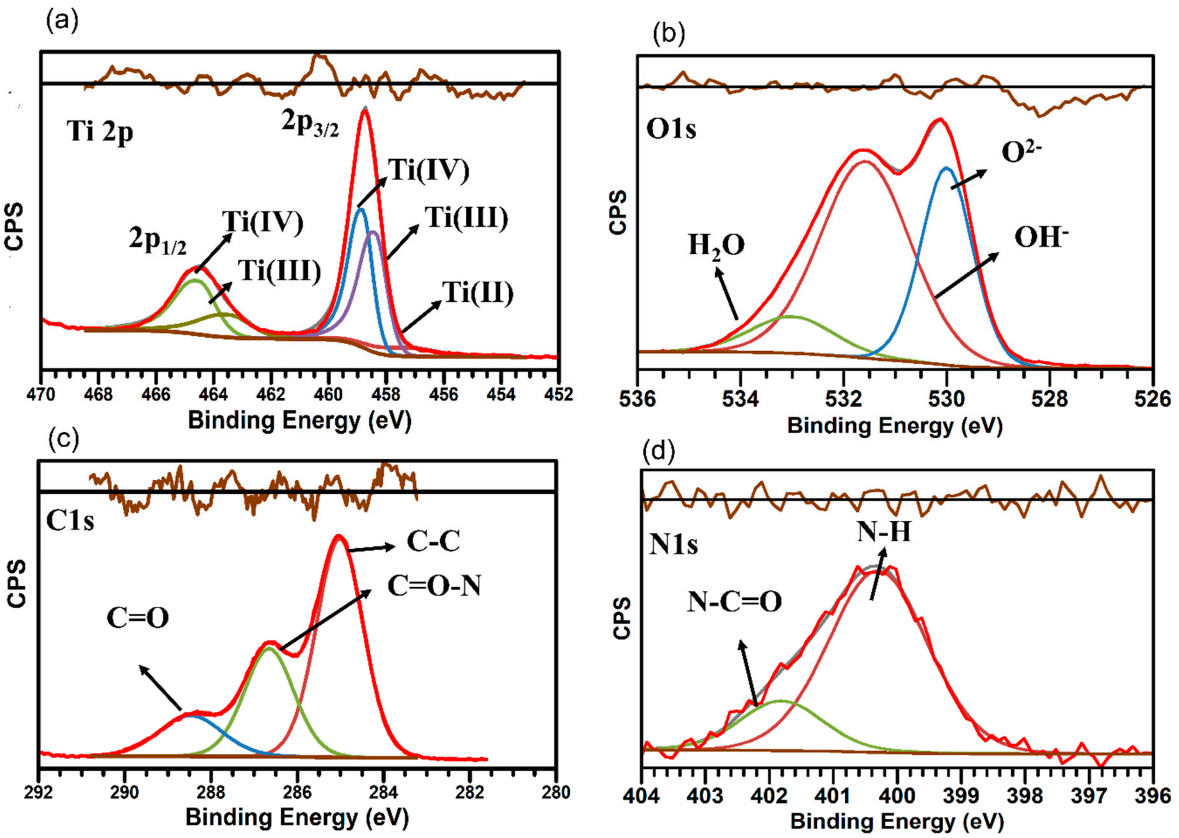

Figure S6: High resolution core-level XP spectra of a) Ti2p, b) O1s, c) C1s and d) N1s registered for a bare Ti6Al4V sample after electrochemical polishing at 9 V for 180 *s*.

6. Raman Characterization

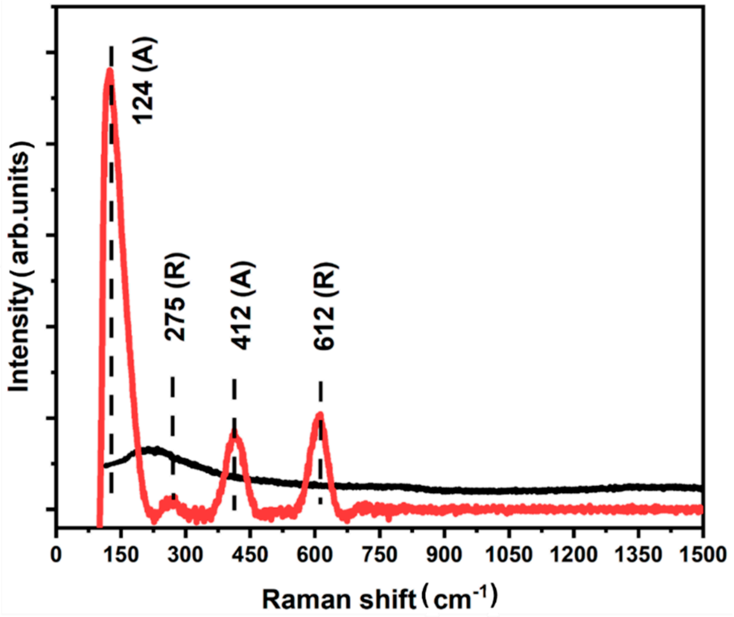

Figure S7: Raman spectra of Ti6Al4V before (black line) and after electrochemical polishing at 9 V for 180 s (red).

The Raman spectra measured for a bare Ti6Al4V substrate before and after electrochemical polishing are shown in Fig. S7. After electrochemical polishing the spectrum shows four characteristic peaks attributed to the rutile and anatase phases of TiO_2. A well-resolved Raman peak is seen at 124 cm^{-1} corresponding to anatase, and three broader contributions found at higher Raman shifts located at around 275 of rutile, 412 of anatase and 612 cm^{-1} of rutile are detected as well. This result confirms that the two phases of TiO_2 are present on the T6Al4V surface after the electrochemical conditioning of the electrode.
